# Bandgap-Tuned 2D Boron Nitride/Tungsten Nitride Nanocomposites for Development of High-Performance Deep Ultraviolet Selective Photodetectors

**DOI:** 10.3390/nano10081433

**Published:** 2020-07-23

**Authors:** Ali Aldalbahi, Rafael Velázquez, Andrew F. Zhou, Mostafizur Rahaman, Peter X. Feng

**Affiliations:** 1 Department of Chemistry, College of Science, King Saud University, Riyadh 11451, Saudi Arabia; mrahaman@ksu.edu.sa; 2 Department of Physics, University of Puerto Rico, San Juan, PR 00936-8377, USA; r.velazquez.vicente@gmail.com; 3 Department of Physics, Indiana University of Pennsylvania, Indiana, PA 15705, USA; fzhou@iup.edu

**Keywords:** 2D boron nitride/tungsten nitride (*BN–WN*) nanocomposite, tunable bandgap, photodetector, fast response

## Abstract

This study presents a fast and effective method to synthesize 2D boron nitride/tungsten nitride (*BN–WN*) nanocomposites for tunable bandgap structures and devices. A few minutes of synthesis yielded a large quantity of high-quality 2D nanocomposites, with which a simple, low-cost deep UV photo-detector (DUV-PD) was fabricated and tested. The new device was demonstrated to have very good performance. High responsivity up to 1.17 A/W, fast response-time of lower than two milliseconds and highly stable repeatability were obtained. Furthermore, the influences of operating temperature and applied bias voltage on the properties of DUV-PD as well as its band structure shift were investigated.

## 1. Introduction

DUV radiation normally can be categorized into three distinct wavelength regions labeled as UVA, UVB and UVC. UVA covers the wavelengths from 400 to 315 nm, while UVB and UVC cover from 315 to 280 nm and from 280 to 100 nm, respectively [1]. During the last several years, much progress has already been made on the development of various visible-blind DUV-PD, based on different wide-band-gap (WBG) nanosemiconductors. The focus of WBG material research for these DUV-PDs was on oxide semiconductors [2,3,4], nitride semiconductors [5,6,7,8], silicon carbide [9,10] and nanodiamond [11,12] materials—as well as different nanocomposites [13,14]. Multilayer heterojunction structures have been widely used [14], and they were basically classified into the metal–semiconductor–metal (MSM) structures, Schottky barriers and p–i–n configurations [15,16]. Various criteria require consideration, including—among others—the optical wavelength of interest, semiconducting material composition and thicknesses, doping element and impurity concentration level, geometric structures or architectures of devices, electrodes and interfaces.

Among differences, MSM structures represent a simple, low-cost device design that is not only related to performance, but also related to easy process in fabrication. The electrical current in MSM is mainly carried by majority charge carriers. The majority carrier current relies on the presence of minority carriers. The minority carriers accumulate at one of the contacts, where they cause additional injection of majority carriers until the minority carriers are recombined. This yields a large photoconductive gain. This is one of the important features for photoconductors. Normally, a large bias voltage is necessary for these PDs [17]. For example, Qian et al. set the bias voltage at 20 V to operate Ga_2_O_3_-based photoconductors [18]; Jin et al. developed ZnO-based ultraviolet photoconductors that operated at a bias voltage of 50 V [19].

A challenge is that long carrier lifetimes in MSM also cause slow response speeds. In most cases, the reported response time was up to several tens of seconds in rise and recovery times [19,20]. In the present work, we report new results of MSM PDs based on emerging semiconductors. Two-dimensional boron nitride/tungsten nitride nanocomposites with controllable bandgap structures were utilized as a sensitive layer. The rationale was that tungsten nitride plays an important role in manipulating composite bandgap. It is closely related to the carrier density and carrier life times that directly affect device gain and response speed. Consequently, very short response times were achieved. Due to the high responsivity to deep-UV emission and insensitivity to sunlight, newly fabricated solar-blind photodetectors could provide a wide range of applications, including ozone damage detection, jet engines related the missile, rocket monitoring and flame detection, combustion monitoring, chemical analysis, NASA space research and astronomy. Many of these applications require detectors capable of operating in high-temperature harsh environments. Si and other narrow bandgap semiconductors are not able to carry out those jobs.

## 2. Experimental Details

The good performance of DUV-PDs relies on their material properties and the detector structural design. Composite 2D BN–WN was fabricated using a pulsed laser-produced plasma deposition technique. A complete explanation of this technique can be found in our previous study [21]. In brief, a high-power CO_2_ laser beam was focused onto BN target in a low (~80 mTorr) pressure chamber. The power density of laser beam on the target was around ~10^8^ W/cm^2^. Tungsten nitride was created using a thermal vapor technique. The BN and WN ratio was controlled at the 4.0 level. Si wafers with oxidized silicon surface were used as substrates placed 4 cm away from the target. The deposition temperature in substrate was around ~500 °C, and the duration for deposition was 20 min. Then, a low-temperature argon plasma source was created at 200 mTorr pressure and utilized to treat nanocomposite layer for 20 min before fabrication of PD devices. The morphologies of nanocomposites were examined using a scanning electron microscope (SEM) and a transmission electron microscope (TEM). Physical properties were characterized with micro-Raman scattering spectroscopy. Finally, the nanocomposite-material-based photodetectors were constructed and assessed.

It should be pointed out that a long minority carrier lifetime resulted in enhancement of photocurrent, but also in slowing the response speed. A basic tradeoff involving the induced photocurrent strength and the response speed of a detector seems inevitable. Usually, the minority carrier time in doped material relies on the capture rate for holes and electrons at the recombination center. It is a function of the doping element concentration. Therefore, nanocomposite ingredients in the present work would be controlled in order to achieve a fast response time, while not to sacrifice too much in photocurrent gain. The obtained data clearly show that the nanocomposites minimized such tradeoff and it displayed an anticipated balance for the DUV-PD’s response speed and light-induced photocurrent strength.

## 3. Results and Discussion

### 3.1. Basic Characterization of Nanocomposite Materials

**Figure 1:**
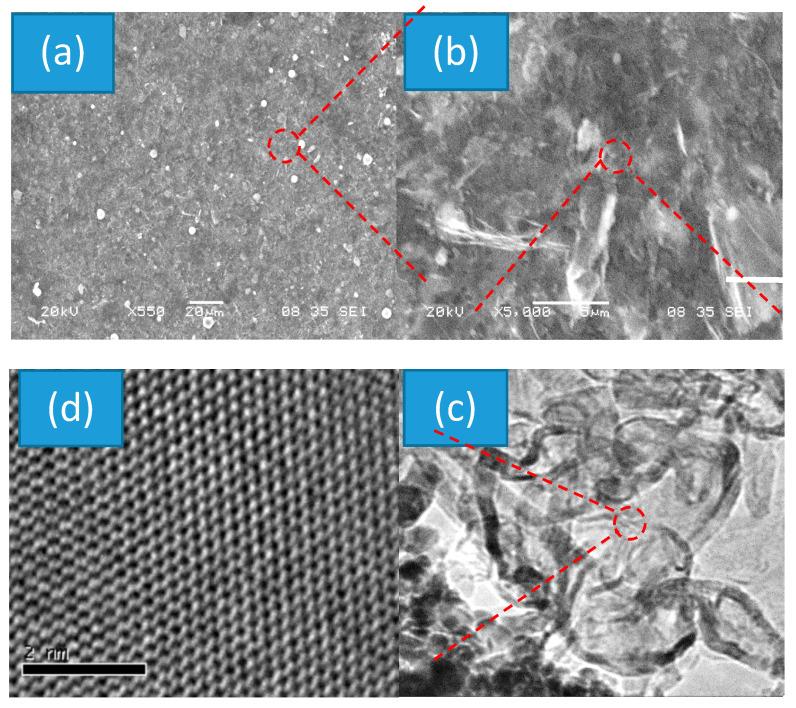
(**a**,**b**) SEM, (**c**) TEM and (**d**) HRTEM images of nanocomposite with different magnifications.

Figure 1a shows an SEM image of the nanocomposite prepared on wafer substrate. From the direct view, it is observed that the obtained film has quite rough surface. The color of the sample appears gray and dull. The SEM image of the composite shows that it is mainly consisted of a large number of small pieces, which are uniformly distributed on the entire substrate surface (1 × 2 cm^2^) area. At high magnification, one could find that the tiny pieces are related to nano sheets and nano ripples, and these are randomly distributed within the nanocomposite as shown in Figure 1b. Average size of each continuous sheet is about a few square micrometers. Figure 1c shows TEM image of the nanocomposite film. It is observed that either sheets or particles were at nanoscale level. Transparent property of sheets could be easily identified. EDS data indicate that the nanosheets were related to BN and the nanoparticles were related to WN material.

In order to understand its good crystalline structures, a single sheet was selected from the samples for HRTEM characterizations. Figure 1d shows its HRTEM image, from which it is observed that each atomic layer consists of a large number of highly ordered boron and nitrogen atom arrays. The period is around 0.22 nm. B_3_ N_3_ rings or two-dimensional benzene-like structures are clearly visible. Neither the N–N bond nor B–B bond was observed.

**Figure 2:**
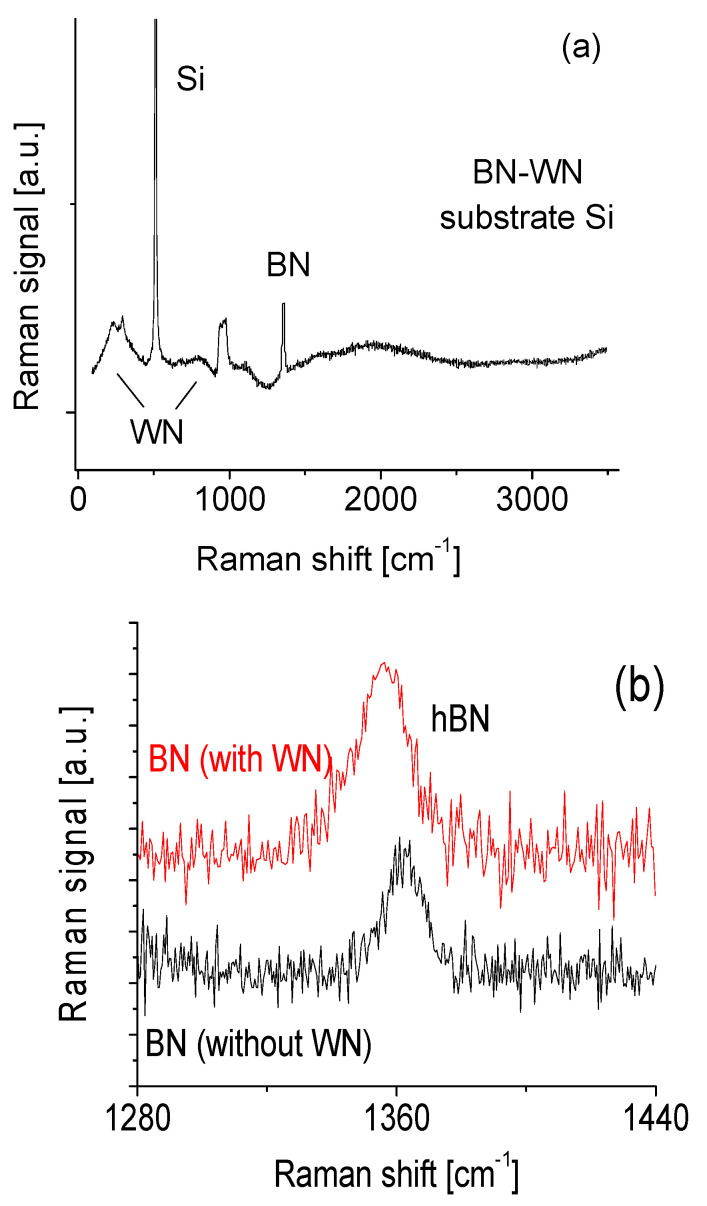
(**a**) Raman scattering spectrum of BN–WN (boron nitride/tungsten nitride) nanocomposite and (**b**) comparison of Raman spectra from BN–WN and BN materials.

Raman spectroscopy was applied to evaluate the nanocomposites, and the results are shown in Figure 2a. The band situated at around 750–850 cm^−1^ was assigned to W–N stretching modes; whereas the band situated at around 250 cm^−1^ was attributed to W–N bending modes. The band at 200–300 cm^−1^ has a stronger Raman signal than that one at ~800 cm^−1^. This is different from that of WO [22,23,24]. In general, tungsten nitride forms together with little tungsten oxide. This is confirmed with EDS measurements. The oxygen is mainly from residue gas in the chamber during nanocomposites synthesis. Detailed discussion of spectral changes from WO to WO_x_N_1−x_ and to WN can be found in previous work [25].

A Raman peak at 1355 cm^−1^ directly corresponds to the active E_2g_ mode of BN [8,26]. This narrow Raman spectral line suggests that the present deposition technique could yield high quality BN composites. Background noise was possibly because of a short accumulation time in Raman measurement. Either to choose higher laser power density or a slower sweep speed during sweeping wave number in Raman measurement would be helpful to obtain a better signal-to-noise ratio.

Three phenomena were observed from the magnified spectral line as shown in Figure 2b after comparing nanocomposite and binary BN Raman spectra. The first is that the Raman active E_2g_ mode with the hexagonal phase shifts from 1363 cm^−1^ for BN to 1355 cm^−1^ for BN–WN. The second is that the Raman spectral line of BN–WN appears to have a slightly asymmetric profile, and the third is that the profile of Raman spectral line of nanocomposite is obviously broadened. These phenomena probably indicated existence of a little defect concentration or contamination. This is in good agreement with our previous results [27]. Generally, the mechanism for the formation of crystalline BNNSs relies heavily on the selected method of synthesis. In the present case, laser heat-driven mechanical exfoliation dominates as the main process in the formation of high-quality crystalline BNNS [27]. Nevertheless, appearance of intense, narrow hBN peak in Raman spectrum indicates the formation of high quality crystalline BN that dominates nanocomposite film.

### 3.2. Fabrication of BN–WN-Based Prototype and Characterizations of Its Basic Electrical Properties

**Figure 3:**
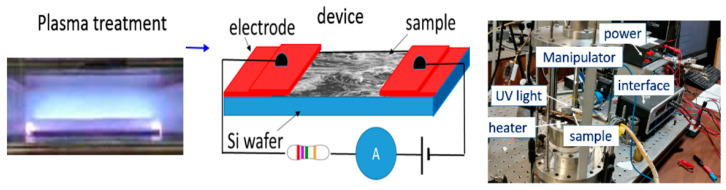
Process flow for nanocomposite-based deep UV photo-detector (DUV-PD) fabrication.

In the present investigation, we extended our previous work on the use of binary BN semiconductor as sensing layer in PD by exploring, synthesis of emerging nanocomposites applied to MSM-based DUV-PDs. The process flow of the prototype fabrication is detailed in Figure 3. Briefly, a low temperature argon plasma source was utilized to treat nanocomposite’s layer for 20 min and then ~100-nm-thick Cu electrodes were deposited at the two ends of the sensing layer. The space between the two Cu electrodes is 150 µm and the width of the same is 4 mm so that total exposed area is 0.6 mm^2^. The sample was connected to a simple electric circuit to form a prototypic DUV PD for the detection of different deep UV radiations.

A planar structure of electrodes was employed in order to optimize the photo-electron collection near to the surface. The sensing layer consists of a large amount of BNNSs, and the thickness of the layer was around 3–5 µm obtained from cross section of SEM image. No precise thickness can be obtained because BNNSs were heavily overlapped one another with random distributions, resulting in a high roughness of the surface. This is very different from a solid thin film.

**Figure 4:**
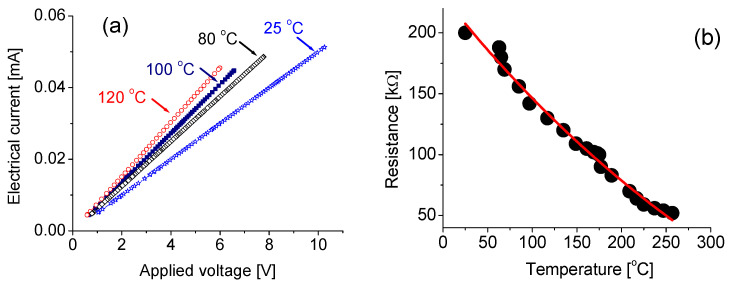
(**a**) Current–voltage (I–V) properties of the DUV-PD at different temperature, (**b**) resistance of active layer as a function of temperature.

The current–voltage (I–V) properties at different operating temperatures were assessed at the homemade test station [28]. The measurements were performed under open-air atmospheric conditions. Figure 4a shows typical current–voltage properties of the prototype operating at 25, 80, 100 and 120 °C, respectively. Linear current–voltage curves are clearly visible, indicating that it is a true Ohmic contact at the interface between the active layer and the electrodes in the prototype.

Variations of temperature slightly affected the electrical properties. As seen in Figure 4b, the resistance of nanocomposites varied from 200 kΩ to 160 kΩ and to 130 kΩ following temperature change from 25 to 80 and to 120 °C, respectively.

### 3.3. Bias Voltage Effect on Response to Light Radiation

**Figure 5:**
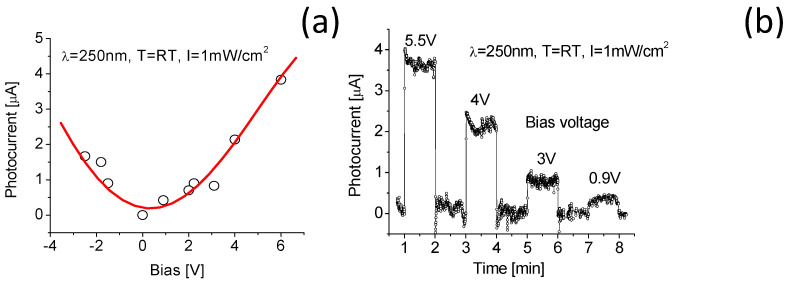
(**a**) Response of the DUV-PD as a function of the bias voltage magnitude; (**b**) time-dependent responses at different applied bias voltages with a fixed 250-nm light radiation intensity and operating temperature.

Before characterization of spectral responses to light radiations, a bias voltage effect was evaluated. Figure 5a shows the induced photocurrent of the nanocomposite-based photodetector as a function of bias voltage where operating temperature and 250 nm radiation intensity remained unchanged. Figure 5b shows the corresponding time-dependent responses at selected biases. High bias voltage yields a high photocurrent or a large response rate (the ratio between the photocurrent output and the light radiation power input). As seen from Figure 5, the induced photocurrent at bias voltage of 5.5 V is about 9 times as large as that at bias voltage of 0.9 V. At the same bias voltage magnitude, the light induced photocurrent under the forward bias voltage is nearly the same value as that under the reverse bias voltage. This is directly attributed to the fact that the prototypic DUV-PD has an MSM structure. The MSM structures represent a simple, low-cost device design. The electrical current in MSM is mainly carried by majority charge carriers. The majority carrier current relies on the presence of the minority carriers. The minority carriers accumulate at one of the contacts, where they would cause additional injection of majority carriers until the minority carriers are recombined. It yields a large photoconductive gain. This is one of the important features for MSM photoconductors. Their main limitations include relatively large dark current (since a bias voltage is required). Therefore, signal-to-noise ratio is relatively low.

A slight variation of the stabilities in response signals and baselines was observed at a high bias voltage as detailed in Figure 5b. It is expected that the increase of bias voltage magnitude would avoidably cause a large dark current or noise. Therefore, a slightly low bias voltage would always be applied in the following experiments.

### 3.4. Spectral Responses to UV Radiations

**Figure 6:**
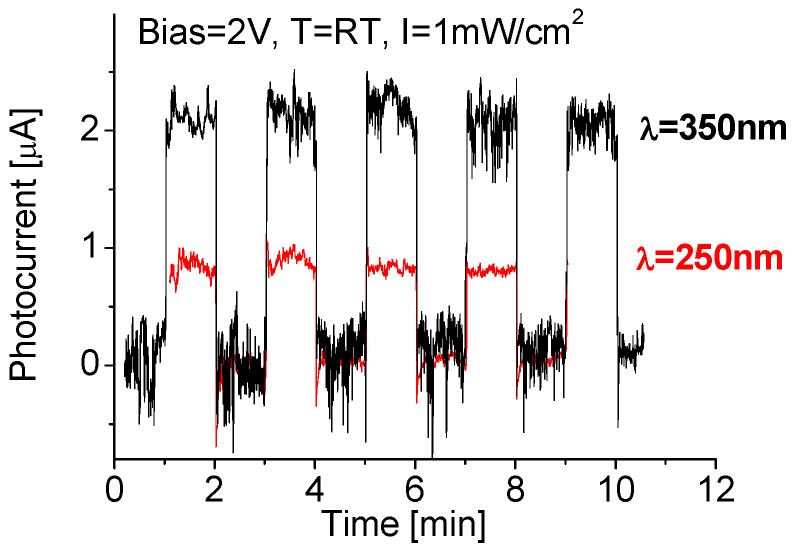
Responses of the device during cyclic tests with 2 min period at RT exposed to 250 nm and 350 nm UV radiations. RT—room temperature; λ: wavelength; B—bias voltage.

Cyclic tests were carried out to understand how much reliable or repeatable a newly fabricated photodetector is. Figure 6 shows the spectral responses of the device during cyclic tests. Good repeatability and stable baseline features were clearly visible. When the cyclic tests were performed at 250 nm radiation, the light induced photocurrent was increased rapidly at initial period, and then reached its maximum. Once the radiation source was turned off, the photocurrent quickly dropped. The induced photocurrent was directly associated with deep ultraviolet light absorption. The maximal photocurrent obtained was 0.84 µA. This is because the exposed area of the PD was—0.6 mm^2^ and the 250 nm radiation power on the active layer was 6 µW, the estimated responsivity (yielded photocurrent to radiation power on the PD) of the PD was around 140 mA/W. This value is more than 100 times larger than that previously reported results obtained either from 2D BN nano-sheets-based or from oxide-semiconductor-based Schottky deep UV photodetectors [29,30,31].

Decreasing the 250 nm UV radiation intensity down to 0.3 mW/cm^2^ on the active layer, the induced photocurrent of the PD drops to 0.53 µA. Further decreasing the 250 nm light radiation intensity to 0.1 mW/cm^2^, the generated photocurrent drops to 0.34 µA. Consequently, a large responsivity up to 560 mA/W was obtained. The phenomenon related to a relatively small value (140 mA/W) of the responsivity at intense (1 mW/cm^2^) deep UV light illumination is possibly due to the saturation effect in light absorption. Such phenomenon was also found in our previous experiments [26].

Interestingly, the fabricated device appears to have the highest response signal to 350 nm light radiation. It is known that the spectral response for the binary BN-based prototypes within the wavelengths ranging from 185 nm to 550 nm has a peak around 200–250 nm [26]. The red spectral shift for BN–WN is due to intrinsic behavior of the nanocomposite as well as its complex and multifactor nature. The best way for measurement of the bandgap shift is to use spectrometers. As an option, Mendoza’s model can also be used [32], with which it is found that the bandgap of the binary BN nanomaterial is around 5 eV (~250 nm); whereas the nanocomposite has a bandgap shift down to 3.5 eV. As seen that 1 mW/cm^2^ 350 nm radiation yielded an induced photocurrent up to 2.2 µA. This is almost 2.6 times as large as that exposed to 250 nm radiation at the same intensity. As radiation intensities decrease from 1.0 to 0.3 and to 0.1 mW/cm^2^, the induced photocurrent decreases from 2.2 to 1.4 and to 0.7 µA; accordingly, the obtained responsivity is 0.33, 0.78 and 1.17 A/W, respectively.

As seen that a large photoconductive gain is a very important feature for MSM photoconductor. The obtained high value of responsivity could also be attributed to a synergistic effect because the nanocomposite material BN–WN was used. This is very different from previous work where most were constructed solely from one material. Composites or multistructure-based materials showed significantly higher efficiency than conventional prototypes constructed solely from one material when tested under identical experimental conditions, suggesting a synergistic effect between the two components.

### 3.5. Time Response

**Figure 7:**
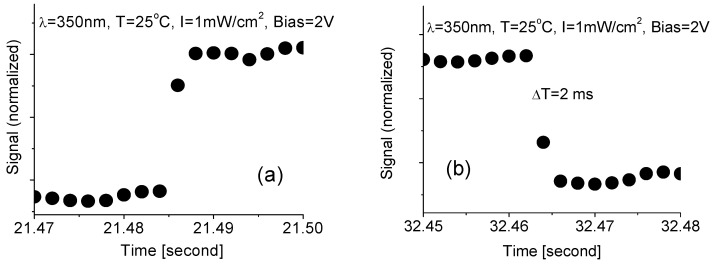
(**a**) Response and (**b**) recovery time of the DUV-PD exposed to 350-nm light radiation at intensity of 1 mW/cm^2^, room temperature and 2-V bias voltage.

In order to analyze response time, a high-resolution interface was utilized to measure the time response of the nanocomposite-based DUV-PD. Typical results are shown in Figure 7, from which the response and recovery times were estimated around 2 ms.

The theoretical limit for the photodetector speed depends on the transit time of electrons and holes in the device, the carrier diffusion and the carrier multiplication process in the BN–WN as well as the circuit time constant. The present prototype should have a fast response speed. This is because the improvement of BN–WN in crystalline quality reduces the recombination and then reduces the response time. Therefore, it can be expected that the real response and recovery times of DUV-PD are shorter than they appear. This is because of the delay in reaching the full intensity after turning on the UV lamp and residual photo-luminescence of the UV light source. The best way for precise measurement of response time is to use a pulsed DUV laser beam or an aligned DUV light beam with an optical chopper System. Unfortunately, this equipment is not available in our laboratories.

### 3.6. Temperature Effect

**Figure 8:**
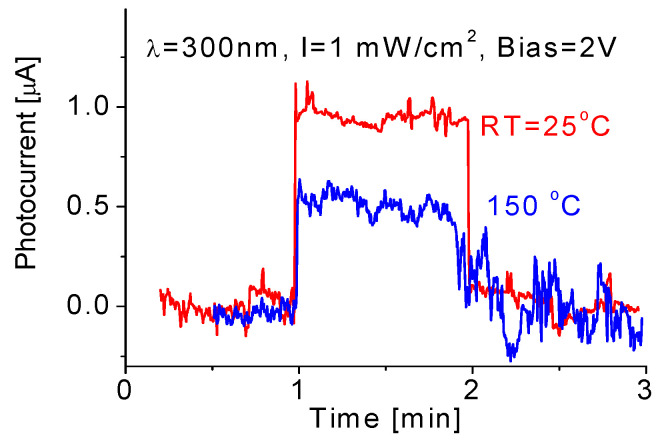
Thermal effect on the responses of the prototypic DUV-PD to 1 mW/cm^2^.

It was found that the variation in operating temperature significantly affected the performance of the prototype exposed to 300 nm radiation as shown in Figure 8. At 25 °C, either response time or recovery time was around few milliseconds. Once the temperature increased to 150 °C, the response time remained nearly unchanged, but the recovery time became longer, around few hundred milliseconds. It is also clear that high operating temperature would result in an intense noise signal and relatively low induced photocurrent. In other words, the obtained photocurrent drops from 0.9 to 0.5 µA following operating temperature increase from room temperature to 150 °C.

A similar phenomenon was also found from the DUV-PD exposed to 250 nm UV radiation, where the response signal or induced photocurrent dropped from 0.84 to 0.24 µA following temperature increase from room temperature to 150 °C. From these above experimental data, we could conclude that even though operating temperature was up to 150 °C, the prototype still ran well with stable repeatability.

## 4. Conclusions

Even though syntheses of nanocomposite materials are extremely easy and fabrication of this prototype is simple and cost-effective, experiments demonstrated that the newly designed prototype has very good performances including fast response time, high photocurrent, good baseline stability and repeatability. A maximal responsivity up 1.17 A/W and response time lower than two milliseconds were achieved. The bandgap shift associated with nanocomposite ingredients directly affected selective spectral responses.

It was concluded that operating temperature notably affected the performance of BN–WN composite-based DUV-PD. When the temperature increased to 150 °C, the induced photocurrent decreased by 30%. The recovery time became slightly longer, but the response time remained unchanged and the prototype still worked well with clear response signal and good responsivity.
